# Oxygen saturations of medical inpatients in a Malawian hospital: cross-sectional study of oxygen supply and demand

**DOI:** 10.15172/pneu.2012.1/208

**Published:** 2016-07-02

**Authors:** Hywel-Gethin Tudur Evans, Nadia Mahmood, Duncan G. Fullerton, Jamie Rylance, Andrew Gonani, Stephen B. Gordon, Kevin Mortimer, Theresa J. Allain

**Affiliations:** 12 Queen Elizabeth Central Hospital, Blantyre, Malawi; 62 Anaesthetics Department, Withybush General Hospital, Fishguard Rd, Haverfordwest, SA612PZ UK; 22 Dept Respiratory Medicine, University Hospital Aintree, Liverpool, UK; 32 Malawi-Liverpool-Wellcome Trust Clinical Research Programme, UK; 42 Liverpool School of Tropical Medicine, UK; 52 College of Medicine, Blantyre, Malawi

**Keywords:** oxygen saturations, oxygen concentrators, pneumonia, tuberculosis, Malawi, Africa

## Abstract

Oxygen is a World Health Organisation listed essential drug yet provision of oxygen in developing countries often fails to meet demand. The aim of this study was to evaluate the need for supplementary oxygen against oxygen delivery capacity at a large teaching hospital in Malawi. A cross-sectional study of all adult medical inpatients and assessment of oxygen provision over a 24-hour period was conducted. 144 patients were included in the study, 14 of whom met local and international criteria for oxygen therapy (oxygen saturations of <90%). Four were receiving oxygen. Of the 8 oxygen concentrators available, only 4 were functional. In conclusion, we identified a need for oxygen that was greater than the supply.

## 1. Introduction

Oxygen is a World Health Organisation (WHO) listed essential drug and it has an important role in the treatment of a wide range of conditions. There is international concern that oxygen provision in developing countries fails to meet demand [1].

Queen Elizabeth Central Hospital (QECH) is a large teaching hospital in southern Malawi which delivers both district level care and tertiary referral services. There are 200 adult medical beds over three wards. The patient population at QECH is very similar to many centres throughout Africa; around 80% of in-patients are infected with Human Immunodeficiency Virus, they are young (mean age 35 years) and over one third present with an acute respiratory illness. The nursing staff to patient ratio is often one nurse to 30 patients. Oxygen is delivered via oxygen concentrators. World Health Organisation guidelines and QECH’s local policy suggest that supplemental oxygen should be provided if patient oxygen saturations (SpO_2_) are <90% [2, 3].

The aim of this survey was to assess the need for supplemental oxygen and its provision.

## 2. Materials and methods

### 2.1. Oxygen need

We conducted a cross-sectional study of SpO_2_ in all medical inpatients over a 24-hour period. SpO_2_ measured below 90% (using a Contec CMS50DL Pulse Oximeter — Contec Medical Systems Co Ltd, USA) were considered abnormal and requiring oxygen. Respiratory rate (RR), patient age and clinical diagnosis were also recorded.

### 2.2. Oxygen provision

**Figure 1:**
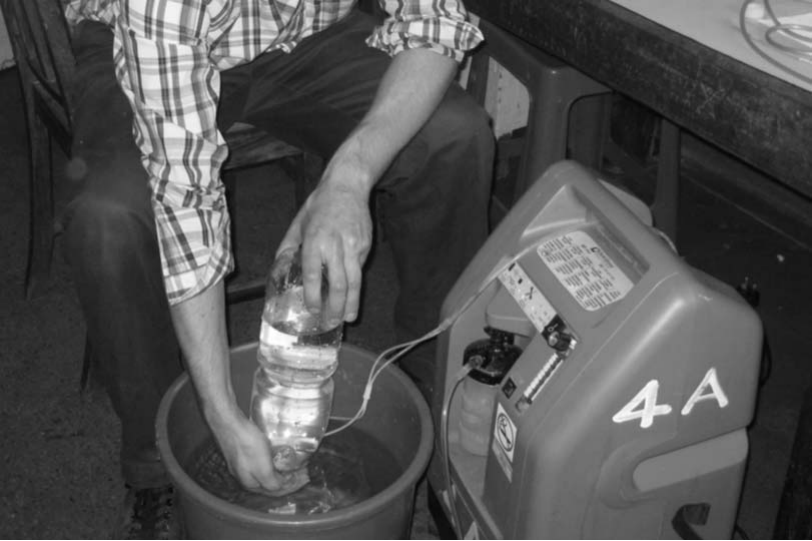
Photograph demonstrating technique used to assess oxygen flow from an oxygen concentrator.

Amongst those patients requiring supplemental oxygen, we assessed if oxygen concentrators were available to them, and if they were functioning adequately. The concentration of oxygen being delivered by each concentrator was measured using a Maxtec MAXO_2_ Oxygen Analyser (Maxtec, USA). Oxygen flow was calculated by measuring the time taken to displace two litres of water from a plastic drinks container held under an underwater seal (Figure 1).

### 2.3. Inclusion and exclusion criteria

All adult medical in-patients were included in the study. Excluded were patients who had not yet been assessed by a doctor or who were not at their bedsides.

### 2.4. Ethical approval

The study was conducted with both departmental and ethics committee approval from Malawi College of Medicine Research and Ethics Committee. Ethics committee approval reference number: COMREC/10. Verbal consent for SpO_2_ to be measured was sought and clinically relevant abnormalities were communicated to the patient’s medical team.

## 3. Results

We assessed 144 of 181 patients (37 patients did not meet inclusion criteria). The mean age was 35 years (SD 14). The most common diagnosis was pulmonary and disseminated tuberculosis (31 and 25 patients, respectively). Twelve patients had pneumonia, 4 a pleural effusion or an empyema and 2 patients had a clinical diagnosis of *Pneumocystis jiroveci* pneumonia. The remaining patients had non-respiratory diagnoses.

**Figure 2:**
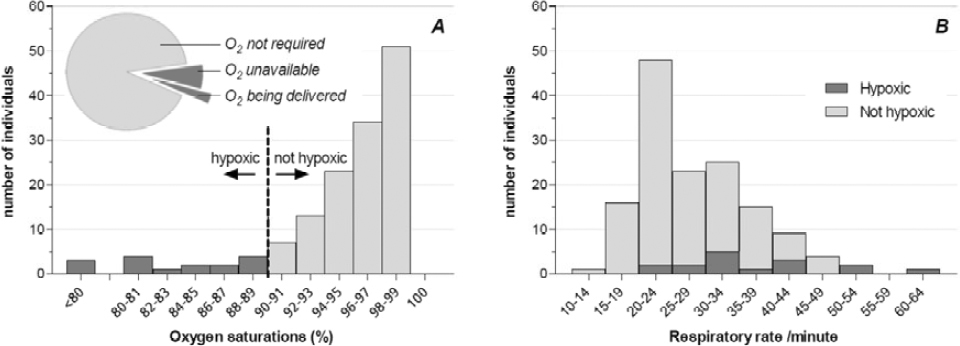
Histograms showing the number of individuals within specific ranges of SpO_2_ (A) and RR (B). Pie chart (2A inlay) showing the majority (71%) of patients who required oxygen were not receiving it.

SpO_2_ values ranged from 53% to 100% with a median of 96% (Figure 2A). Fourteen individuals (9%) had oxygen saturations <90% and, therefore, met the criteria for oxygen therapy.

Of these 14 hypoxic patients, 4 (29%) were receiving oxygen therapy (Figure 2A inlay). Few patients had a normal RR (range 12–60, mean 28, Figure 2B). There was little correlation between RR and SpO_2_ (Figure 2B).

We identified 3 patients whose hypoxia had not previously been recognised.

Eight concentrators were present in the department; 4 of which functioned appropriately. Three provided oxygen at <60% of the indicated flow rate. One of these 3 delivered oxygen at a concentration of <60%. One concentrator did not function.

## 4. Discussion

Our study has shown that over one third of patients were admitted for an acute respiratory illness and that hypoxia was not identified 21% of the time; less than a third of patients who required supplemental oxygen were receiving it. Half of the concentrators did not function adequately. Even if all the concentrators were functional, demand would still exceed supply capacity by 75%.

Pulse oximetry is superior to clinical signs alone in detecting hypoxia [4]. In the context of hypoxia secondary to an acute respiratory illness, supplemental oxygen saves lives; in resource poor settings, pneumonia deaths can be reduced by 35% [5]. Oxygen concentrators provide the cheapest and most consistent source of oxygen in such healthcare facilities where power supplies are reliable [6].

Supplemental oxygen should be prioritised according to need. Importantly, this first requires the recognition of hypoxia and secondly, the availability of a functional oxygen supply. Recognition of hypoxia within the department is limited by the fact that the department has no pulse oximeters of its own despite persistent procurement requests to the Ministry of Health (MoH), Malawi. The use of pulse oximeters is sporadic as, although some clinicians have their own, most cannot afford this.

The medical department is allocated eleven oxygen concentrators by the MoH. However, functioning units are persistently fewer. Those available range in age from one to five years and each one is used virtually continuously. Despite a scheduled program of maintenance, the machines are in practice rarely adequately serviced, not least, due to the need for their near consistent use and the unavailability of spare parts. The lack of access to a properly functioning oxygen supply together with the lack of pulse oximetry to aid the recognition of hypoxia compounds the disparity between the patients’ need for oxygen and its provision in this setting.

A strength of our study is that it formally quantified the extent of a well-recognised but frequently neglected problem. However, as a 24-hour cross-sectional study it only provides a brief snapshot and is open to random error as well as information and selection bias. Malawi essentially has three seasons: hot and wet, hot and temperate. As a result, there are seasonal variations in the number of admissions as well as the severity of illness with a consequent impact on the need for oxygen. The survey was conducted in May, at the start of the temperate season, when admission numbers are near the monthly average. Further snapshots throughout the year would enhance our understanding of how well the need for oxygen is being met.

Although we were able to assess the functionality of the oxygen concentrators e.g. the flow and concentration of oxygen that they generated, using basic equipment, a lack of resources limited our ability to fully assess the cause for equipment malfunctioning. This information would be of value as it could potentially demonstrate the benefit of even minimal servicing e.g. cleaning filters and replacing broken tubing. Nonetheless, our primary objective was to assess the magnitude of the disparity between the need for and availability of oxygen, rather than to pinpoint specific causes for equipment malfunctioning.

We hope that our data provides evidence to enable appropriate allocation of resources which, in turn, will increase the availability of pulse oximeters and oxygen concentrators. We anticipate that other hospital departments in similar settings will benefit from undertaking similar assessments.

The Global Pulse Oximetry Project, a partnership including the WHO and the World Federation of Societies of Anesthesiologists, aims to increase uptake of pulse oximetry in operating theatres in low-income countries by making available low-cost pulse oximeters. The expansion of this project to include Emergency and High Dependency Units would be most welcomed.
